# Cranial morphology of captive mammals: a meta-analysis

**DOI:** 10.1186/s12983-021-00386-0

**Published:** 2021-01-23

**Authors:** Leila Siciliano-Martina, Jessica E. Light, A. Michelle Lawing

**Affiliations:** 1 Interdisciplinary Program in Ecology & Evolutionary Biology, Texas A&M University, College Station, TX 77843 USA; 2 Department of Biology, Texas State University, San Marcos, TX 78666 USA; 3 Department of Ecology and Conservation Biology, Texas A&M University, College Station, TX 77843 USA

**Keywords:** Captivity, Zoo, Cranial morphology, Meta-analysis

## Abstract

**Background:**

Captive facilities such as zoos are uniquely instrumental in conservation efforts. To fulfill their potential as bastions for conservation, zoos must preserve captive populations as appropriate proxies for their wild conspecifics; doing so will help to promote successful reintroduction efforts. Morphological changes within captive populations may be detrimental to the fitness of individual animals because these changes can influence functionality; thus, it is imperative to understand the breadth and depth of morphological changes occurring in captive populations. Here, we conduct a meta-analysis of scientific literature reporting comparisons of cranial measures between captive and wild populations of mammals. We investigate the pervasiveness of cranial differences and whether cranial morphological changes are associated with ecological covariates specific to individual species, such as trophic level, dietary breadth, and home range size.

**Results:**

Cranial measures of skull length, skull width, and the ratio of skull length-to-width differed significantly between many captive and wild populations of mammals reported in the literature. Roughly half of captive populations differed from wild populations in at least one cranial measure, although the degree of changes varied. Carnivorous species with a limited dietary breadth displayed the most consistent changes associated with skull widening. Species with a more generalized diet displayed less morphological changes in captivity.

**Conclusions:**

Wild and captive populations of mammals differed in cranial morphology, but the nature and magnitude of their cranial differences varied considerably across taxa. Although changes in cranial morphology occur in captivity, specific changes cannot be generalized for all captive mammal populations. The nature of cranial changes in captivity may be specific to particular taxonomic groups; thus, it may be possible to establish expectations across smaller taxonomic units, or even disparate groups that utilize their cranial morphology in a similar way. Given that morphological changes occurring in captive environments like zoos have the potential to limit reintroduction success, our results call for a critical evaluation of current captive husbandry practices to prevent unnecessary morphological changes.

**Supplementary Information:**

The online version contains supplementary material available at 10.1186/s12983-021-00386-0.

## Background

Captive facilities such as zoos are important hubs for in situ and ex situ conservation where animals are often maintained in an effort to preserve species as faithful representatives of their wild counterparts so that captive populations can sustain the functionality and fitness of the species and perhaps, one day, be considered for reintroduction [39, 64, 75, 92, 117]. However, morphological changes occurring in a captive population may reduce the fitness of individual animals given that cranial morphology largely confers functionality [57, 74, 109]. It is therefore crucial to understand the breadth and depth of the morphological changes occurring in captive populations.

The skulls of captive mammals may differ from wild populations in both size and shape (e.g., [46, 88, 110, 120]). Documented differences include changes in the cranial length and width of African lions (*Panthera leo* [43, 46];), sagittal crest height of Amur tigers (*P. tigris* [28];), and mandibular morphology of Japanese macaques (*Macaca fuscata* [50];), traits which are integral for feeding and influence bite force and dietary niche [77, 79, 108, 122]. The relative spread of the zygomatic arch is highly indicative of cranial musculature and functionality, where a wider zygomatic arch implies the presence of enhanced musculature and a stronger bite force often associated with carnivores (e.g., [24, 43]) and gnawing rodents (e.g., [30]). Although morphological changes in captivity have been reported in the literature, the nature (i.e., directionality and magnitude) of cranial differences and the ecological factors that may drive these differences (i.e., ecological covariates) have remained unclear.

The morphological differentiation of captive populations from their wild counterparts may be the result of inbreeding or evolutionary processes (e.g., genetic drift, selection), phenotypic plasticity (the ability of genotypes to display multiple, environmentally dependent phenotypes), or some combination of these factors [27, 33, 64, 113, 121]. Correlations have been found between a decrease in body size and inbreeding among captive wolves [44, 47, 58]. Molecular signatures of inbreeding and genetic drift have been noted among white-footed mice (*Peromyscus leucopus*) maintained in captivity [114, 115]. Lynch & Hayden [63] suggested the cranial changes they observed among farmed American mink (*Mustela vison*) were largely the result of differing selection pressures. Abnormal skull morphology of several captive mammals, including coyotes (*Canis latrans* [24];), African lions (*Panthera leo* [43];), and Japanese macaques (*Macaca fuscata* [50];) have all largely been attributed to phenotypic plasticity.

In captivity, unusual phenotypes, especially of the crania, may be expressed as a plastic response to environmental factors related to novel diet textures [24, 46], nutrient availability [61, 103], or any other factors unique to the captive environment [40, 41, 87]. Cranial responses to a captive environment may be explained by differences in muscle usage, which may impact osteological traits [17, 87, 117, 119]. For example, a soft diet requires less musculature and therefore less mechanical stress is applied to the cranial bones, potentially resulting in a bone whose difference is greater than the variance of the mean wild-type morphotype [17, 46, 117]. Reduced mechanical constraint is also associated with reduced covariation between internal and external cranial morphology [24]. Therefore, a species whose wild diet is composed of particularly tough items may be more prone to morphological changes in captivity if captive diets are softer than what the animal might consume in the wild [24, 46, 51].

In addition to diet texture, the shape of cranial bones may be influenced by other factors related to captivity, such as stereotypic behaviors. Stereotypies are quite common among captive animals, yet they are rare in wild populations [69, 70]. Stereotypies are repetitive behaviors that serve no obvious function [69]; however, they may impact morphology due to the frequent, abnormal muscle usage involved in their performance [28, 43, 88, 99]. Stereotypies can include normal behaviors performed to the point of self-destruction (e.g., licking, grooming, rubbing) as well as head swinging, bar-biting, and pacing [69, 70, 72, 73]. Stereotypic overgrooming, for example, has been correlated with changes in the cranial morphology of captive tigers, where captive individuals display malformed sagittal crests associated with the heightened muscle usage involved in incessant grooming behaviors [28]. Stereotypic behaviors tend to be most common among captive animals with large wild home ranges [19, 56, 71] and those with highly specialized diets or food acquisition behaviors [65, 69, 73].

The degree to which morphology differs in captivity compared to wild populations may vary among species. For example, while African lions tend to show rather drastic, consistent morphological changes associated with an increase in zygomatic breadth [43, 46, 125], house mice (*Mus musculus*) show little morphological change in captivity [23]. Even closely related taxa may differ in the degree of change that they exhibit once in captivity [38, 50, 97], possibly due to species ecology where certain traits may predispose a particular species to a specific captive response. The likelihood of morphological changes occurring in captivity may increase when an animal’s habitat is difficult to replicate (leading to heightened stress behaviors) or when diets are difficult to accommodate [18, 24, 56]. Hypercarnivory (a diet that consists of a minimum of ~ 70% vertebrate prey [25, 109];), for instance, may predispose species to more extreme morphological differentiation in captivity [24, 43]. This is because skull shape is strongly linked to dietary function among wild carnivores [100, 109] and diet in captivity may be drastically different than it is in the wild [51]. Similarly, species that consume large prey have comparatively round skulls, where bowed zygomatic arches and heightened sagittal crests enable enhanced musculature and increased jaw strength [30, 100, 109]. If appropriate diets are not provided, differentiation in cranial morphology may occur in captivity [22, 24, 43, 46].

While the effects of captivity on mammalian cranial morphology are a recurrent theme in morphological research, the design of these studies vary, making it difficult to draw substantive and comprehensive conclusions about the nature of morphological changes occurring in captivity. The literature is generally limited to case studies of single species, several closely related species, or computational models predicting phenotypic trajectories. Here, we use a phylogenetic meta-analysis to examine effects reported in the existing literature and whether there are consistent differences in cranial morphological changes to help identify characteristics of species at the greatest risk of morphological change in captivity. In particular, we focused on studies reporting skull length, skull width, and the ratio of skull length to width, as these traits are intimately linked to cranial size and functionality [31, 60]. The magnitude and directionality of morphological changes among captive populations are expected to vary based on ecological factors, where the largest morphological changes are likely to occur among species whose diets and habitats are particularly difficult to accommodate in captivity (e.g., large home range size, carnivorous, narrow dietary breadth). Given that they may be responding to similar captive stressors, these species are also expected to display similar morphological changes (e.g., wider zygomatic breadth). By examining these hypotheses and developing a more comprehensive understanding of morphological changes that occur in captivity, effects may be reduced with updated husbandry practices to help ensure the long-term maintenance of captive populations and their potential for reintroduction success.

## Results

### Literature search

An exhaustive literature search with key words “zoo”, “captive”, “mammal”, “animal”, “skull”, “cranium”, “morphology”, and “size” revealed 515 potentially relevant publications examining differences in the cranial morphology of a population of captive mammals, of which 17 met the complete search criteria (examining a non-domesticated species in captive populations that have not experienced intentional artificial selection) and included all applicable data required for inclusion in at least one of the size or shape-related trait analyses (Table 1). The dates of the studies included in these analyses ranged from 1894 to 2020; however, methods used in most studies (caliper measurements) have not changed considerably over the past century. In total, these 17 publications included 47 comparative relationships between wild and captive populations (15 shape and 32 size-related variables), across 21 species, representing six mammalian orders. The most well-represented groups included carnivorans (*n* = 6) and primates (*n* = 5), although our meta-analyses also included ungulates (*n* = 4), rodents (n = 4), and two marsupials (Table 1).

**Table 1:** Studies and species included in each meta-analysis

Species	Family	Order	Study	L	W	L:W
*Acinonyx jubatus*	Felidae	Carnivora	[78]	X	X	X
*Bettongia gaimardi*	Potoroidae	Diprotodontia	[95]	X		
*Canis latrans*	Canidae	Carnivora	[24]	X	X	X
*Canis lupus*	Canidae	Carnivora	[120]	X	X	X
*Chlorocebus aethiops*	Cercopithecidae	Primates	[104]	X		
*Dicerorhinus sumatrensis*	Rhinocerotidae	Perissodactyla	[38]	X	X	X
*Equus africanus*	Equidae	Perissodactyla	[37]	X	X	X
*Equus hemionus*	Equidae	Perissodactyla	[37]	X	X	X
*Gorilla gorilla*	Hominidae	Primates	[110]	X	X	X
*Hydrochoerus hydrochaeris*	Caviidae	Rodentia	[2]			X
*Lemur catta*	Lemuridae	Primates	[98]		X	
*Microtus arvalis*	Cricetidae	Rodentia	[7] (captive), [68] (wild)		X	
*Mustela nigripes*	Mustelidae	Carnivora	[5]		X	X
*Mustela nigripes*	Mustelidae	Carnivora	[116]	X		
*Myodes glareolus*	Cricetidae	Rodentia	[8] (captive), [9] (wild)		X	
*Pan troglodytes*	Hominidae	Primates	[110]	X	X	X
*Panthera leo*	Felidae	Carnivora	[43]	X	X	X
*Panthera tigris*	Felidae	Carnivora	[43]	X	X	X
*Peromyscus polionotus*	Cricetidae	Rodentia	[74]			X
*Pongo pygmaeus*	Hominidae	Primates	[110]	X	X	X
*Rhinoceros unicornis*	Rhinocerotidae	Perissodactyla	[38]	X	X	X
*Sminthopsis macroura*	Dasyuridae	Dasyuromorpha	[39]	X		

Meta-analyses include skull length (L), skull width (W), and the ratio of skull length-to-width (L:W). For full citations, please see the reference section

In total, our study consisted of six meta-analyses, including analyses of size (skull length and width) and shape (skull length-to-width) to examine the magnitude and directionality of changes. For each assessment of cranial size and shape, data were analyzed with and without an absolute value applied to the standard effect sizes (a standardized statistic that encodes quantitative data from multiple studies into a common form [62];). Each of the meta-analyses were conducted as independent models with and without the inclusion of ecological covariates (trophic level, dietary breadth, home range size; Table S1), which may influence the degree of morphological differentiation between captive populations and their wild counterparts. Each of our meta-analyses included a slightly different collection of taxa based on taxa composition and their measures reported in each publication (Table 1). Publication bias has the potential to over-inflate the significance of meta-analytic models potentially leading to Type 1 errors, given studies that recover significant results may be more likely to be published [16, 62]. However, we did not detect publication bias in any of our meta-analyses (Fig. S1).

A strong phylogenetic signal was recovered in analyses of directionality associated with skull length (λ=0.89) and skull length-to-width (λ=0.88). Analyses of the magnitude of change did not recover a strong phylogenetic signal (Table 2).

**Table 2:** Pagel’s λ results to detect a phylogenetic signal

Feature	Analysis	λ Mean	λ StDev
Skull Length	Directional	0.89^*^	0.01
	Magnitude	2.2e-4	6.8e10–4
Skull Width	Directional	0.01	0.07
	Magnitude	0.54	0.15
Skull L:W	Directional	0.88^*^	0.01
	Magnitude	0.70	0.01

Mean lambda estimate from 2000 randomly selected phylogenic trees and the standard deviation (StDev) around that mean for skull length, skull width, and skull length-to-width (L:W) analyses. Significant results are indicated in bold and asterisks indicate *p*-value range: 0.01–0.05*

### Morphological changes in captivity

**Fig. 1:**
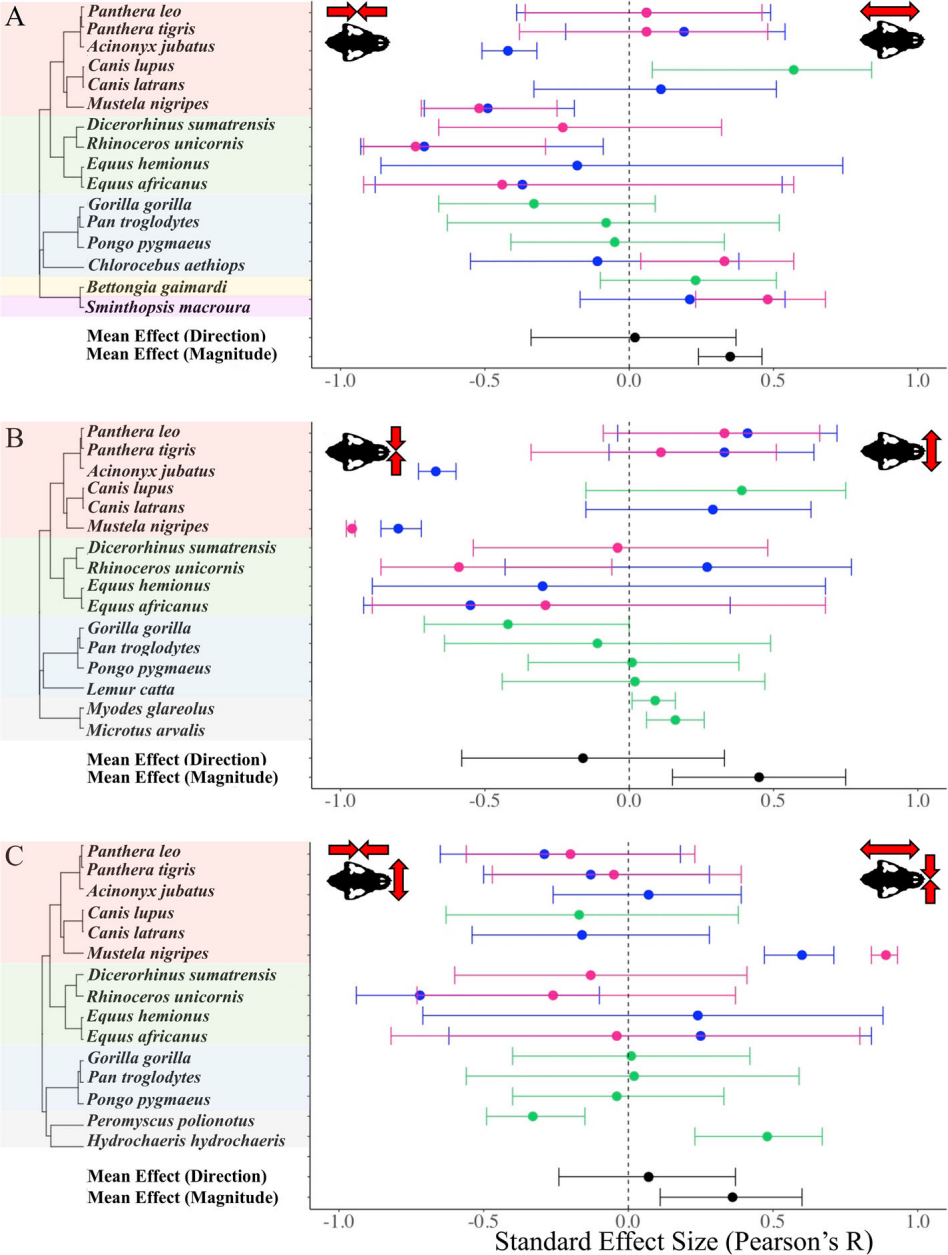
Cranial difference between captive and wild populations of individual mammal species. Forest plot of each meta-analysis for **a**: skull length, **b**: skull width, and **c**: skull length-to-width. Phylogenies shown to the left of each meta-analysis plot were pruned from Upham et al. [105]. Mammalian orders are indicated by color on the phylogeny: red=Carnivora, green=Perissodactyla, blue=Primates, purple=Dasyuromorpha, yellow=Diprotodontia, and gray=Rodentia (see Table 1 for additional taxonomic information). Colors within the plot indicate the sex of the specimens from each study: magenta=females, blue=males, and green indicates a study that used a pooled sample of both sexes. Summary effect sizes for directional analyses as well as analyses of magnitude are indicated in black. Forest plot lines that do not cross the dotted zero line are associated with a significant effect (i.e., the effect is not zero). Red arrows on the skull illustrations indicate the morphology associated with the positive and negative effect sizes on each plot

**Fig. 2:**
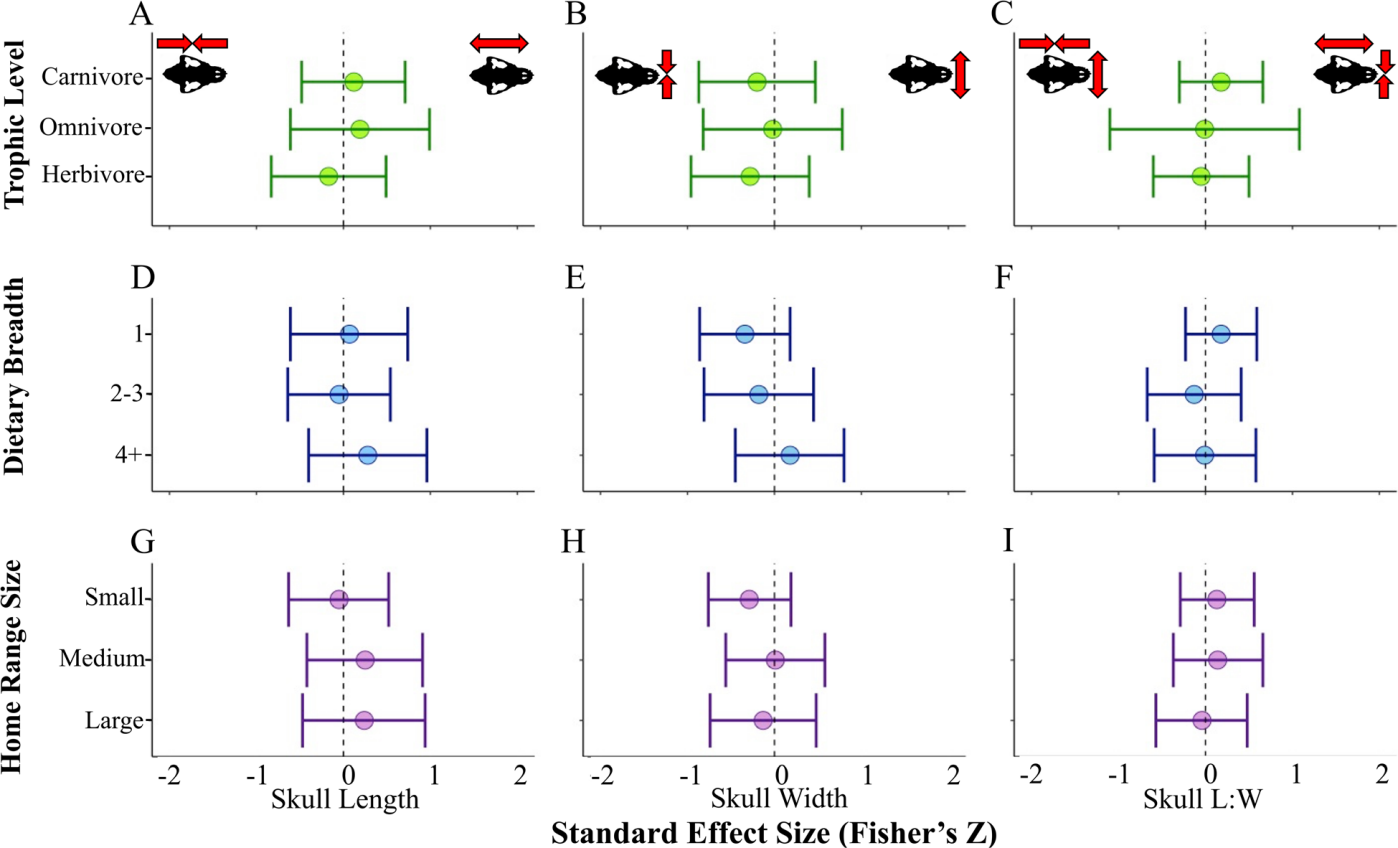
Analyses of directionality associated with ecological covariates. **a**-**c**: trophic level, **d**-**f**: dietary breadth, and **g**-**i**: relative home range size. Columns indicate analyses of skull length (**a**, **d**, **g**), skull width (**b**, **e**, **h**), and skull length-to-width (**c**, **f**, **i**). Forest plot lines that do not cross the dotted zero line are associated with a significant effect. Red arrows on the skull illustrations indicate the morphology associated with the positive and negative effect sizes on each plot

Roughly half of species (11 of 21) displayed distinct trait values in captive populations in at least one of the analyses (and in at least one of the sexes, when more than one sex was reported). Skull length and width changes were often apparent among rodents and carnivores (including species in order Carnivora as well as the striped-faced dunnart, *Sminthopsis macroura*, a carnivorous marsupial; Fig. 1). These changes were generally not detected among primates and were only apparent in the skull length of gorillas and female vervet monkeys (*Chlorocebus aethiops*) (Fig. 1a). Several carnivorous species displayed changes related to an elongation and widening of the skull (e.g., *Canis lupus, Panthera leo, S. macroura*), while others (e.g., *Acinonyx jubatus* and *Mustela nigripes*) displayed the opposite pattern with a shorter, narrower cranium in captivity (Fig. 1a and b). Most rodents included in this study (e.g., *Microtus arvalis*, *Myodes glareolus,* and *Peromyscus polionotus*) displayed a significant increase in cranial width within the captive populations compared to their wild counterparts (Fig. 1b and c), while captive capybara (*Hydrochoerus hydrochaeris*) displayed an elongation and narrowing of the cranium (Fig. 1c). When sex was reported, similar morphological trends were typically displayed between both sexes within species, with some exceptions (e.g., female vervet monkeys, *Chlorocebus aethiops* displayed significantly longer crania in captive populations, although males did not; Fig. 1a). We did not detect a significant relationship between any of the directional changes in cranial morphology observed in captivity and the ecological covariates included in this study (trophic level, dietary breadth, and home range size; Fig. 2).

**Fig. 3:**
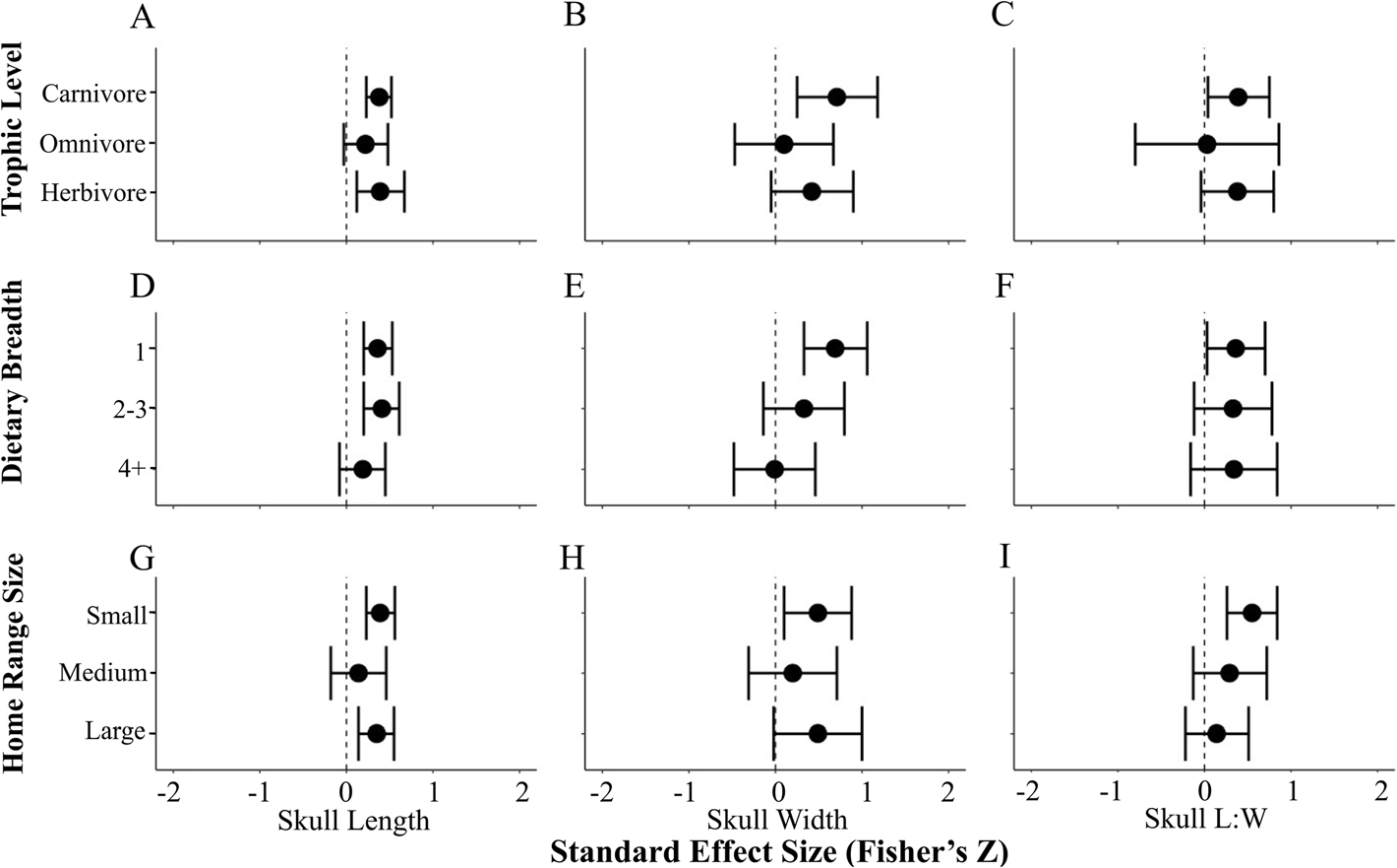
Analyses of magnitude associated with ecological covariates. **a**-**c**: trophic level, **d**-**f**: dietary breadth, and **g**-**i**: relative home range size. Columns indicate analyses of skull length (**a**, **d**, **g**), skull width (**b**, **e**, **h**), or skull length-to-width (**c**, **f**, **i**). Forest plot lines that do not cross the dotted zero line demonstrate a significant difference between captive and wild populations

Phylogenetic meta-analysis models of the magnitude of change detected moderate to strong effects (r) for skull length (r = 0.35, *p* = 1.3e-9***), skull width (r = 0.45, *p* = 3.7e-3**), and skull length-to-width (r = 0.36, *p* = 4.0e-3**; Table 3); 0.3 < r < 0.5 is interpreted as a moderate effect and r > 0.5 is considered a strong effect in the ecological literature [35, 83]. Analyses of magnitude also revealed moderate to strong effects associated with the carnivorous trophic level (skull length, r = 0.38, *p* = 5.7e-7***; skull width, r = 0.71, *p* = 3.0e-3**; skull length-to-width, r = 0.39, *p* = 0.03*; Table 3; Fig. 3a-c), the narrowest dietary breadth (skull length, r = 0.36, *p* = 9.1e-6***; skull width, r = 0.69, *p* = 1.9e-4***; skull length-to-width, r = 0.36, *p* = 0.03*; Table 3; Fig. 3d-f), and the smallest home range size (skull length, r = 0.39, *p* = 2.3e-6***; skull width, r = 0.49, *p* = 0.01*; skull length-to-width, r = 0.55, *p* = 2.0e-4***; Table 3; Fig. 3g-i). Phylogenetic meta-analysis models of the magnitude of skull length also revealed strong effects associated with herbivory (r = 0.39, *p* = 4.7e-3**; Table 3; Fig. 3a) and a moderate dietary breadth (r = 0.41, *p* = 1.2e-4***; Table 3; Fig. 3d).

**Table 3:** Results for all full model and ecological covariate meta-analyses

			Directionality					Magnitude				
			r	SE	Z	LB	UB	r	SE	Z	LB	UB
**Skull Length** =	Full Model		0.01	0.19	0.07	−0.37	0.39	0.35	0.06	6.07^***^	0.24	0.46
	Trophic Level	Carnivores	0.12	0.30	0.38	−0.48	0.71	0.38	0.08	5.00^***^	0.12	0.67
		Omnivores	0.19	0.41	0.47	− 0.61	0.99	0.22	0.13	1.70	−0.33	0.48
		Herbivores	−0.17	0.34	−0.50	− 0.83	0.49	0.39	0.14	2.83^**^	0.12	0.67
	Dietary Breadth	1	0.07	0.34	0.19	−0.61	0.74	0.36	0.08	4.44^***^	0.20	0.53
		2–3	−0.05	0.30	−0.16	− 0.64	0.54	0.41	0.11	3.83^***^	0.20	0.62
		4+	0.28	0.35	0.81	−0.40	0.96	0.19	0.14	1.34	−0.08	0.45
	Home Range	Small	−0.05	0.29	−0.18	−0.63	0.52	0.39	0.08	4.72^***^	0.23	0.56
		Medium	0.25	0.34	0.73	−0.42	0.91	0.14	0.16	0.86	−0.18	0.46
		Large	0.24	0.36	0.66	−0.47	0.94	0.35	0.11	3.31^***^	0.14	0.55
**Skull Width** =	Full Model		−0.19	0.19	−0.98	− 0.57	0.19	0.45	0.15	2.91^**^	0.15	0.75
	Trophic Level	Carnivores	−0.20	0.34	0.59	−0.87	0.47	0.71	0.24	2.99^**^	0.25	1.18
		Omnivores	−0.02	0.41	−0.05	− 0.82	0.78	0.10	0.29	0.35	−0.47	0.67
		Herbivores	−0.28	0.35	−0.82	−0.96	0.40	0.42	0.24	1.74	−0.05	0.90
	Dietary Breadth	1	−0.34	0.26	−1.28	−0.86	0.18	0.69	0.19	3.73^***^	0.33	1.06
		2–3	−0.18	0.32	−0.57	− 0.81	0.45	0.33	0.24	1.40	−0.13	0.80
		4+	0.18	−0.32	− 0.55	−0.45	0.80	−0.01	0.23	−0.04	− 0.48	0.46
	Home Range	Small	−0.29	0.24	−1.19	−0.76	0.19	0.49	0.20	2.44^*^	0.10	0.88
		Medium	0.01	0.29	0.03	−0.56	0.58	0.20	0.26	0.78	−0.31	0.71
		Large	−0.13	0.31	−0.41	−0.74	0.48	0.49	0.26	1.87	−0.02	1.00
**Skull L:W** =	Full Model		0.07	0.16	0.45	−0.25	0.39	0.36	0.13	2.85^**^	0.11	0.60
	Trophic Level	Carnivores	0.18	0.25	0.72	−0.30	0.66	0.39	0.18	2.16^*^	0.04	0.75
		Omnivores	−0.01	0.56	−0.02	−1.10	1.08	0.03	0.42	0.07	−0.80	0.86
		Herbivores	0.05	0.28	−0.17	−0.60	0.50	0.38	0.21	1.78	−0.04	0.80
	Dietary Breadth	1	0.18	0.21	0.86	−0.23	0.59	0.36	0.17	2.14^*^	0.03	0.70
		2–3	−0.13	0.27	−0.47	− 0.67	0.41	0.33	0.23	1.47	−0.12	0.78
		4+	−0.01	0.30	−0.02	−0.59	0.58	0.34	0.25	1.34	−0.16	0.84
	Home Range	Small	0.13	0.22	0.62	−0.29	0.56	0.55	0.15	3.72^***^	0.26	0.84
		Medium	0.14	0.26	0.54	−0.37	0.66	0.29	0.22	1.34	−0.13	0.72
		Large	−0.04	0.25	−0.16	−0.57	0.48	0.14	0.19	0.77	−0.22	0.51

Cranial morphological differences between captive and wild mammals assessing directionality and magnitude on skull length, skull width, and skull length-to-width (L:W). Results report the mean value from 2000 randomly selected phylogenic trees from Upham et al. [105] where r represents the summary effect of the model, SE represents the standard error, Z represents the Z-Statistic of the model, and LB and UB represent the lower and upper bounds, respectively, of the confidence interval. Ecological covariates include trophic level, dietary breadth, and home range sizes. Significant results are indicated in bold and asterisks indicate *p*-value range: 0.01–0.05*, 0.001–0.01**, 0–0.001***

## Discussion

Captive facilities such as zoos can provide an environment for breeding and maintenance of threatened or endangered species that may otherwise exist in perilous conditions [4, 10, 21]; however, questions regarding the morphological differentiation of captive populations have caused concern that these facilities may not preserve species as appropriate proxies for the wild population. The results of our phylogenetic meta-analyses of magnitude indicate that although directionality of the morphological changes occurring in captivity varied between taxa, the overall magnitude of standard effect size was consistently different from zero. This suggests the mammals examined in our study display size and shape changes in captivity, but they do not all differ in the same way. Although analyses that use absolute values have been shown to inflate model significance and create artificially narrow confidence intervals [84, 91], we chose to analyze these data because the direction of change in traits appeared inconsistent, yet there were many trait-species combinations that had no 95% confidence overlap with zero, indicating change, regardless of direction, may be indicative of populations in captivity. Applying absolute values to standard effect sizes neutralizes directionality and can therefore help interpret whether there are any general effects of captivity, particularly when extreme positive and negative values exist in the dataset.

The differentiation in cranial morphology between captive and wild populations of rodents, carnivorans, and the carnivorous marsupial *S. macroura* may be related to the well-developed temporalis muscles that these species possess [30]. Temporalis muscles enhance jaw strength at the anterior portion of the skull, which translates to enhanced incisor gnawing strength among rodents and increased force distributed to the canine teeth of carnivorous mammals [30]. The presence of an enlarged temporalis muscle requires a common set of morphological specializations including a wider zygomatic arch (i.e., a wider skull [30];). Species that do not heavily rely on anterior jaw strength, such as most herbivores and omnivores, typically display a narrower zygomatic breadth (i.e., a narrower skull [30];), as we saw in this study (Fig. 2b). The cranial shapes observed in captivity may seem counterintuitive, where captive specimens of several species display wider skulls than their wild counterparts, with documented instances of correspondingly enhanced cranial musculature [87] and bite force [32]. This seemingly contradictory result may be related, at least in part, to the changes in muscle usage that occur in captivity [40, 41, 87]. While the pressure to capture prey and flee predators may be removed in captivity, certain captivity-specific tasks, such as the performance of stereotypic behaviors and the processing of novel diets may also increase muscle usage and may explain the morphological differences observed in this study [40, 41, 87, 98]. Similarly, wild diets may constrain cranial shapes to maintain optimum functionality for processing and capturing prey items, particularly among species with highly specialized diets [2, 32, 60, 109]. In captivity, the absence of these natural selection pressures may lead to a greater distribution of mean trait values or a loss in the covariation of cranial modules [24, 74, 76].

Trophic level, dietary breadth, and home range size were all associated with changes in morphology in captive animals. As noted above, carnivorous species tended to display morphological change in captivity; however, trophic level was broadly categorized with only carnivorous, omnivorous, and herbivorous groupings. A more detailed examination of dietary categories including subcategories associated with folivory, insectivory, or granivory, for example, may provide a more nuanced exploration of the role trophic level plays in the morphological change observed in captivity. Species with the largest home range sizes in the wild frequently display heightened stereotypies in captivity [19, 56, 71] and possible subsequent morphological change compared to wild populations, although some of the most distinct morphological shifts we observed were associated with species that inhabit the smallest home range sizes (e.g., *M. nigripes*; Fig. 1). However, it may be that this finding is more strongly associated with trophic levels given that some of the species that displayed the largest morphological changes are carnivorous and also have some of the smallest home range sizes.

The specific morphological changes associated with captivity varied within mammalian orders. For example, while the majority of rodents included in this study (*Microtus arvalis*, *Myodes glareolus*, and *Peromyscus polionotus*) displayed a wider cranium in captivity (Fig. 1b and c), the zoo population of capybaras (*Hydrochaeris hydrochaeris*) displayed the opposite trend, with a narrower, more elongated cranium in captivity. Capybara are distinct from the other rodent species included in this study in several ways. With an average body weight of roughly 50 kg, capybara are the largest rodent species and the only members of Family Caviidae included in this study (all other rodents in this study are members of Family Cricetidae; Table 1). Unlike many rodent species, capybara also have a semi-aquatic lifestyle and as selective grazing herbivores that consume primarily grass, they fill a dietary niche that is more similar to cattle than it is to many other rodent species [11, 49]. The lifestyle and dietary differences between capybara and the other rodent species included in this study may help to explain the opposing morphological trends that we observed. Likewise, while captive gray wolves (*Canis lupus*) displayed longer crania in captivity, captive cheetahs (*Acinonyx jubatus*) and black-footed ferrets (*Mustela nigripes*) displayed notably shorter crania, although all three species are members of Order Carnivora. The reasons for this difference within a relatively close taxonomic unit are unclear but may be related to the extensive population bottlenecks experienced by the latter species [80, 116]. While certain morphological changes occurring in captivity have been attributed to selection [64, 115] or phenotypic plasticity [40, 87] for traits that may be advantageous in the captive environment, species with especially depleted genetic lines may display less favorable morphological traits. However, this topic requires further investigation into the mechanisms that drive these changes and the relative favorability of these traits in captivity.

Although studies examining the morphological effects of captivity often assess exclusively adult specimens, mammalian cranial morphology may differ significantly between older and younger adult specimens, where certain skull proportions, such as facial length, width, and mandibular length may continue to change throughout an adult animal’s lifetime [3, 106, 107]. Within human populations, for example, the cranial elements of a person at age 30 can differ significantly from those at age 80 [3, 48]. Animals maintained in captivity may experience a longer lifespan than those living in the wild [26, 53] and be subject to additional morphological change. It is unclear how these age-related morphological changes may influence differentiation of captive and wild populations. Specific age information was largely unavailable in the studies included in these meta-analyses, although nearly all specimens were designated as adults (aside from two studies that did not report age information and one study that included three ‘nearly mature’ specimens, see the methods section), thus we were unable to consider this topic more closely. Future research on the effect of age on morphology should examine whether the increased lifespan frequently found in captivity may act as a confounding factor in the morphological differentiation observed between these populations.

Captive animals often have well-documented histories, exist in highly controlled environments, and, in some cases, represent the largest accessible populations of rare or endangered species [13, 14, 101, 104] and are thus ideal for biological research, especially studies focusing on morphological changes over time compared to wild populations. However, our findings suggest that certain specimens from captive populations should be preferentially avoided in morphological research, particularly carnivorous species and rodents, which can display distinct morphological shifts in captivity. In contrast, captive primates and other omnivorous species appear to show negligible shifts in cranial size and shape. This supports the findings of Bello-Hellegouarch et al. [14], whose geometric morphometric study of great ape scapula found similarly limited differences between wild and captive populations. Our findings suggest that researchers examining some species, or anatomical regions unlikely to be impacted by captivity, need not avoid captive specimens in future morphological studies.

## Conclusions

Changes in cranial morphology of captive mammals may impact dietary function and limit the conservation potential of captive populations [6, 74, 92, 114, 117]. Although differences in cranial morphology of captive mammals has long been recognized, the nature and commonality of those differences have been poorly understood. The findings of our phylogenetic meta-analyses suggest that differences in mammalian cranial morphology occur in captivity, but the nature and magnitude of those differences often varies among species. The overall magnitude of these differences implies that further investigation within individual species and at higher taxonomic levels is warranted to better understand how and why cranial morphology changes in captivity compared to wild mammal populations, especially studies examining the evolutionary mechanisms of these morphological changes. As captive facilities such as zoos become increasingly responsible for the long-term survival of threatened and endangered species [21, 112, 118], developing an understanding of the morphological changes occurring in captivity will be essential to avoid these effects in the future.

## Methods

We conducted phylogenetic meta-analyses to examine differences in cranial morphology of captive mammals compared to their wild counterparts documented in previously published literature. We focused on three traits: skull length and skull width (traits associated with size), and the ratio of skull length-to-width (a trait associated with shape).

### Literature search and meta-analysis

We conducted an exhaustive search of the literature using search functions in Web of Science, Google Scholar, and PQDT Open. Searches were conducted using the key terms, ‘zoo’ or ‘captive’, ‘mammal’ or ‘animal’, and ‘skull’, ‘cranium’, ‘morphology’ or ‘size’ and were completed in August 2020. Additional studies were located by searching the reference sections of literature on the topic. Literature searches were refined to only include studies which 1) provided comparative size and/or shape data of captive and wild mammals, 2) assessed non-domesticated captive species (as described in [72], following the species listed in [59, 123]), and 3) assessed captive populations that had not experienced intentional artificial selection.

Captive facilities included zoos, laboratories, or other breeding centers. Animals that were bred for specific traits (e.g., farm populations bred for size, laboratory colonies bred for particular attributes) were excluded from these analyses. Efforts were made to assess exclusively adult animals; exceptions to this included three of six captive female *Dicerorhinus sumatrensis* specimens, which were suggested to be ‘nearly mature’ [37] and two studies that did not specify the age of the specimens that were analyzed [5, 37]. In studies examining multiple age groups (e.g. [2]), only the data from the adult categories were included. We downloaded data associated with the publications and when relevant data were unavailable with the publication, we contacted the corresponding author with a request to share available data. If data could not be obtained, these studies were removed from analyses (*n* = 3). All analyses followed the Preferred Reporting Items for Systematic Reviews and Meta-Analyses (PRISMA) statement guidelines, which provides recommendations for the inclusion of studies in meta-analyses, as well as parameters for data extraction [82].

We conducted six phylogenetic meta-analyses, including analyses of size (skull length and width) and shape (skull length-to-width) to examine both the magnitude and directionality of changes for mammalian populations in captivity compared to their wild counterparts. We describe the ratio of skull length-to width as a shape using the most general definition of the term in which the ratio of two linear measures provides an approximation of a structure’s shape [55, 85]. Each of our meta-analyses included a different collection of taxa based on the measures reported in each publication (Table 1). We used standard effect sizes (a standardized statistic that encodes quantitative data from multiple studies into a common form [62];) and the absolute value of standard effect sizes in our meta-analyses. We also conducted meta-analyses with the inclusion of ecological covariates (trophic level, dietary breadth, home range size; Table S1).

To assess publication bias, the tendency for significant results to be disproportionally published [15, 93], we used funnel plots that display the distribution of standard effect sizes with corresponding variances. Asymmetry in a funnel plot is indicative of publication bias, whereas an unbiased sample will produce a relatively conical pattern of points. Egger’s regression (mixed-effect meta-regression model) was used to assess asymmetry in each funnel plot [29, 86, 102].

### Standard effect size

Data including sample size, mean, and standard deviation or standard error were extracted from each study and used to calculate a standard effect size for each variable (including skull length, skull width, and skull length-to-width; Table S2). When no variance measures were provided [37, 38], the prognostic method, a conservative estimate of missing variance terms, was applied to estimate missing standard deviations (Table S2) using the sample size and variance data available in the other studies included in the dataset (see the following for a review of these methods: [66, 67]). To calculate the corresponding standard effect size of shape ratios, pooled standard deviations were calculated based on 10,000 permutations using the sample size, mean, and standard deviation of both linear measures. When necessary, measurements were extracted from publication figures using MorphoJ ([54]; Table S2), which provides an estimate of the x- and y- coordinates of every point in a scatterplot. These values were then used to calculate the mean and standard deviation of the measures associated with captive and wild populations in the study. To assess the effects across studies, Pearson’s correlation coefficient (r) was calculated as a measure of the standard effect size [20] and converted to Fisher’s Z, a normality transformation typically applied to meta-analyses [1, 12, 96].

Both traditional (*n* = 15) and geometric morphometric (*n* = 2) studies were assessed in these analyses (Table S2). Linear measures found in traditional morphometric studies were used to estimate skull length and skull width. To derive shape variables from traditional morphometric studies, ratios of linear measures (i.e., skull length to skull width) were taken. Skull shapes were extracted from geometric morphometric studies using principal component (PC) scores reported in the publications. As a rigid rotation, principal component analyses preserve the covariation between specimens, where PC scores represent independent axes of shape variation [81, 124] and were used exclusively in the meta-analysis of cranial shape (skull length-to-width). Specific shapes represented by each PC was determined from publication text and figures depicting morphology (e.g., thin-plate splines). When necessary (only for *Peromyscus polionotus*, [74]) the extracted PC scores were multiplied by − 1 to preserve the relative skull length-to-width relationship between the captive and wild populations described in the manuscript text, given the relative ordination of the PCA provided in the publication.

### Ecological covariates

We assessed the ecological covariates trophic level, dietary breadth, and home range size (assessed in wild populations) to evaluate the ways in which species ecology may be associated with changes in morphology in captive populations (Table S1). These ecological data were derived from the open-access PanTHERIA dataset [49]. Trophic levels included carnivorous, omnivorous, and herbivorous, which are broadly defined in the PanTHERIA dataset by the presence or absence of vertebrate or non-vertebrate prey in an animal’s diet. A carnivorous species is defined by its nearly exclusive consumption of either vertebrate or invertebrate prey, herbivorous species are defined by not consuming any prey items, and omnivorous species are defined by their consumption of mixed dietary categories. Dietary breadth accounts for the number of dietary categories consumed by a species and ranges from one to eight in the PanTHERIA dataset. For our analyses, dietary breadth was parsed into three categories, including species that consume items from a single dietary category, those consuming two to three dietary categories, and those consuming four or more dietary categories. Lastly, home range sizes in the wild were recorded as the average area inhabited by a species (km^2^); these values were converted to categorical variables (small, 0.01–5 km^2^; medium, 11–30 km^2^; and large, 55–160 km^2^) based on the distribution of these values in this dataset. Home range sizes were estimated from additional sources when data were not available for a given species in PanTHERIA, including Asiatic wild asses (*Equus hemionus* [36];), vervet monkeys (*Chlorocebus aethiops* [45];), European pine voles (*Microtus subterraneus*), and stripe-faced dunnarts (*Sminthopsis macroura*) whose estimated home range size was inferred to be less than 0.1 km^2^ based on the data available for other members of the genus in PanTHERIA.

### Phylogenetic non-Independence

We pruned phylogenetic trees inferred by Upham et al. [105] to species present in each phylogenetic meta-analysis using *picante* [1, 52]. Pagel’s λ was used to assess phylogenetic signal of standard effect size for each of the variables we evaluated [34, 89, 90] by assessing 2000 randomly selected phylogenies from Upham et al. [105] with *geiger* [42]. Pagel’s λ results are reported as the mean and standard deviation of all iterations (Table 3). A strong phylogenetic signal was recovered in analyses of skull length and skull length to width (see Results and Table 3). Thus, these analyses were conducted using phylogenetic comparative methods to account for evolutionary non-independence in our meta-analyses ([34, 35]; Table 3).

Multi-variate phylogenetic meta-analyses were conducted with *Metafor* [1, 93, 111]. Studies included in our meta-analyses reported values for single sexes, individual values for each sex, or pooled samples of both sexes. Thus, we incorporated a random effect variable of sex in meta-analytic models, in addition to other typical random effects variables of study and species [12, 111]. The ‘species’ random effect accounted for the uneven inclusion of species in the analyses and the phylogenetic covariance was specified by the correlation matrix [111]. The ‘study’ random effect accounted for the variation across individual studies. Each phylogenetic meta-analysis was conducted using 2000 randomly selected phylogenies from Upham et al. [105] and reported as mean and standard deviations of those iterations. Phylogenetic covariance was calculated for each phylogeny as described in Adams [1]. All analyses were conducted in R version 3.6.1 [94].

## Supplementary Information

**Additional file 1.**

## Data Availability

All aggregated data analyzed during this study are included in this published article and its supplementary information files. Any additional data are available upon request to the corresponding author.
