# Supplementary material for: Cranial morphology of captive mammals: a meta-analysis
Source: Front Zool. 2021 Jan 23;18:4. doi: 10.1186/s12983-021-00386-0 (PMC7825229; doi:10.1186/s12983-021-00386-0)

Supplemental Data

Table S1: Ecological covariate categories applied to species in this study as found in PanTHERIA (Jones et al., 2009). Trophic levels include carnivores, omnivores, and herbivores. Dietary breadth refers to the number of dietary items consumed by the species, either 1, 2-3, or 4+ items. Home range sizes ranged from small (S), to medium (M), to large (L). See Table 1 for taxonomic information each species.

| **Species** | **Trophic**  **Level** | **Dietary Breadth** | **Home Range**  **Size** |
| --- | --- | --- | --- |
| *Acinonyx jubatus* | Carnivore | 1 | L |
| *Bettongia gaimardi* | Herbivore | 2-3 | S |
| *Canis latrans* | Carnivore | 1 | M |
| *Canis lupus* | Carnivore | 1 | L |
| *Chlorocebus aethiops* | Omnivore | 4+ | S |
| *Dicerorhinus sumatrensis* | Herbivore | 2-3 | M |
| *Equus asinus* | Herbivore | 1 | L |
| *Equus hemionus* | Herbivore | 1 | M |
| *Gorilla gorilla* | Omnivore | 2-3 | S |
| *Hydrochoerus hydrochaeris* | Herbivore | 4+ | S |
| *Lemur catta* | Omnivore | 4+ | S |
| *Microtus arvalis* | Herbivore | 2-3 | S |
| *Mustela nigripes* | Carnivore | 1 | S |
| *Myodes glareoulus* | Omnivore | 4+ | S |
| *Pan troglodytes* | Omnivore | 4+ | M |
| *Panthera leo* | Carnivore | 1 | L |
| *Panthera tigris* | Carnivore | 1 | L |
| *Peromyscus polionotus* | Herbivore | 2-3 | S |
| *Pongo pygmaeus* | Omnivore | 4+ | S |
| *Rhinoceros unicornis* | Herbivore | 2-3 | S |
| *Sminthopsis macroura* | Carnivore | 2-3 | S |

Table S2: Data extracted from studies. Type refers to geometric or traditional morphometric analyses. Source is the section of the publication from which the data were extracted. Data were reported as a mean and variance measure. Variance measures included standard deviations (StDev), standard error (SE), or the prognostic method (Prog), which was used to estimate the standard deviation. See Table 1 for taxonomic information each species.

| **Species** | **Type** | **Study** | **Source** | **Data Reported** |
| --- | --- | --- | --- | --- |
| *Acinonyx jubatus* | Traditional | Meachen et al., 2020 | Table 2 | Mean, StDev |
| *Bettongia gaimardi* | Traditional | Rose, 1984 | Table 3.10 | Mean, StDev |
| *Canis latrans* | Traditional | Curtis et al., 2018 | Table 4 & Supplement | Mean, StDev |
| *Canis lupus* | Traditional | Wolfgramm, 1894 | Table 2 | Mean, StDev |
| *Chlorocebus aethiops* | Traditional | Turner et al., 2016 | Table 2 & 3 | Mean, StDev |
| *Dicerorhinus sumatrensis* | Traditional | Groves, 1982 | Table 1 | Mean, StDev |
| *Equus asinus* | Traditional | Groves, 1966 | Table 1 | Mean, Prog |
| *Equus hemionus* | Traditional | Groves, 1966 | Table 1 | Mean, Prog |
| *Gorilla gorilla* | Traditional | van Velzen, 1967 | Figures 5, 7, 9 | Mean, StDev |
| *Hydrochoerus hydrochaeris* | Geometric | Aeschbach et al., 2016 | Supplement & author | Mean, StDev |
| *Lemur catta* | Traditional | Selvey, 2018 | Table 3.1 | Mean, StDev |
| *Microtus arvalis* | Traditional | Balčiauskienė, 2007 (captive),  Markov et al., 2012 (wild) | Table 1 / Table 1 | Mean, StDev |
| *Mustela nigripes* | Traditional | Antonelli, 2015 | Table 3.1 & 3.2 | Mean, StDev |
| *Mustela nigripes* | Traditional | Wisely et al., 2002 | Table 3 | Mean, SE |
| *Myodes glareoulus* | Traditional | Balčiauskienė, 2007b (captive),  Balčiauskiene & Balčiauskas, 2009 (wild) | Table 1 / Figure 3b | Mean, StDev |
| *Pan troglodytes* | Traditional | van Velzen, 1967 | Figures 5, 7, 9 | Mean, StDev |
| *Panthera leo* | Traditional | Hartstone-Rose et al., 2014 | Supplement | Mean, StDev |
| *Panthera tigris* | Traditional | Hartstone-Rose et al., 2014 | Supplement | Mean, StDev |
| *Peromyscus polionotus* | Geometric | McPhee, 2004 | Figure 4 | Mean, StDev |
| *Pongo pygmaeus* | Traditional | van Velzen, 1967 | Figures 5, 7, 9 | Mean, StDev |
| *Rhinoceros unicornis* | Traditional | Groves, 1982 | Table 4 | Mean, StDev |
| *Sminthopsis macroura* | Traditional | Guay et al., 2012 | Table 1 & author | Mean, StDev |

Figure S1: Funnel plots exploring publication bias in the dataset for each meta-analysis conducted. Asymmetry was assessed with Egger’s regression. Significant results are indicated in bold and asterisks indicate *p*-value range: 0.01-0.05*, 0.001-0.01**, 0-0.001***.


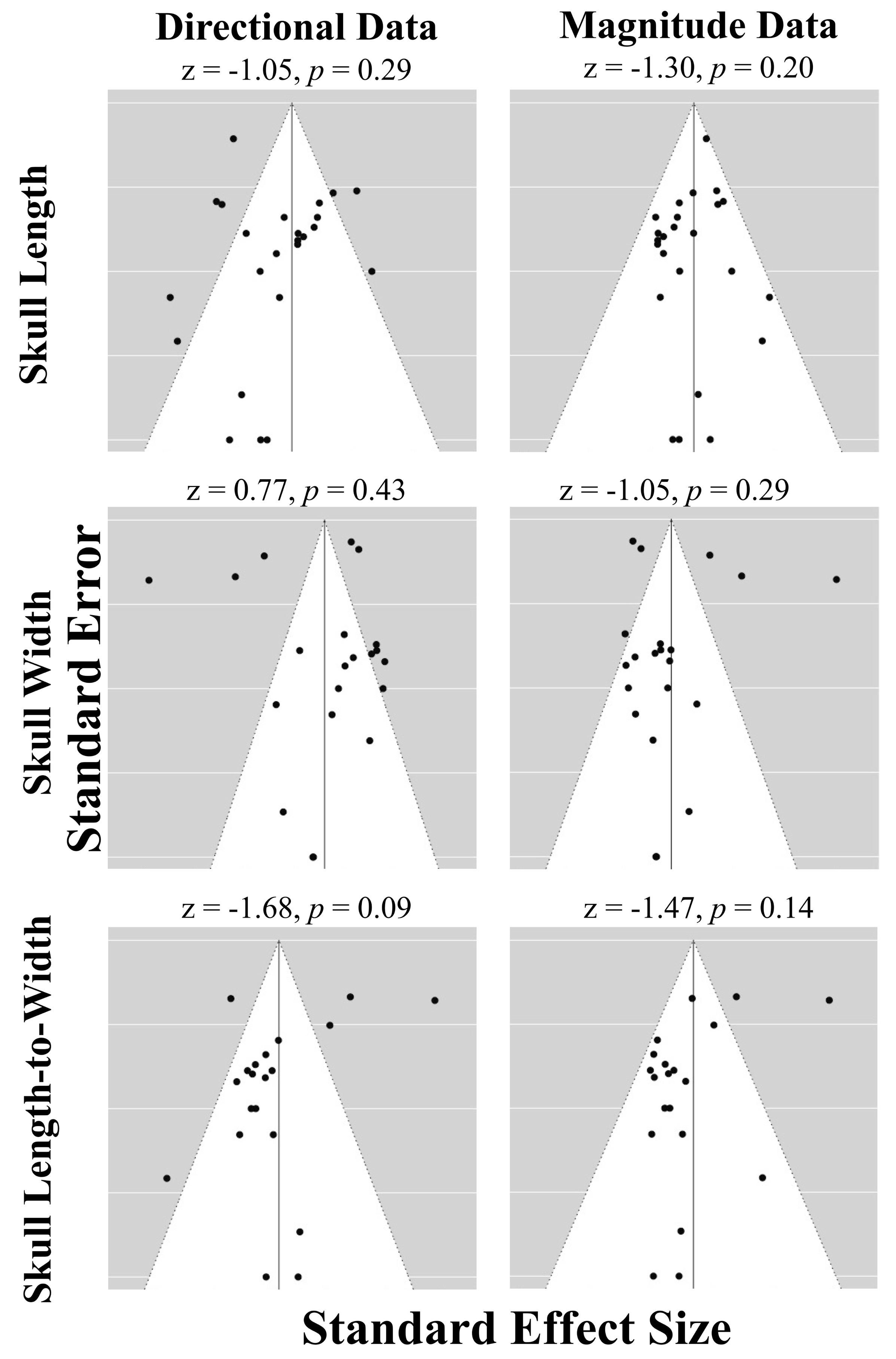

Supplement: Supplementary file 1 — Additional file 1. [file 12983_2021_386_MOESM1_ESM.docx]
